# Selenoprotein P-1 (SEPP1) as an Early Biomarker of Acute Kidney Injury in Patients Undergoing Cardiopulmonary Bypass

**DOI:** 10.31083/j.rcm2305170

**Published:** 2022-05-11

**Authors:** Davide Bolignano, Federica Jiritano, Mariateresa Zicarelli, Patrizia Pizzini, Sebastiano Cutrupi, Michele Andreucci, Alessandra Testa, Domenica Battaglia, Belinda Spoto, Pasquale Mastroroberto, Giuseppe Filiberto Serraino, Giuseppe Coppolino

**Affiliations:** ^1^Nephrology and Dialysis Unit, Magna Graecia University, 88100 Catanzaro, Italy; ^2^Cardiac Surgery Unit, Magna Graecia University, 88100 Catanzaro, Italy; ^3^Institute of Clinical Physiology, National Research Council (CNR), 89124 Reggio Calabria, Italy

**Keywords:** selenoprotein-p1, acute kidney injury, cardiopulmonary bypass, biomarker

## Abstract

**Background:**

Acute Kidney Injury (AKI) is a frequent, dangerous complication in patients undergoing cardiopulmonary bypass (CPB) with oxidative stress playing a crucial role. In this pilot study we evaluated the possible role of the selenoprotein-p1 (SEPP1), a circulating, anti-oxidant selenium transporter, as a predictive biomarker of AKI in this population setting.

**Methods:**

Circulating SEPP1 was measured in the blood of 45 patients before surgery and at 4 h, 8 h and 12 h after CPB by Enzyme-Linked Immunosorbent Assay (ELISA).

**Results:**

SEPP1 increased from 69 [IQR 39–85] to 3263 [IQR 1886.2–5042.7] ng/mL (*p* for trend <0.0001). AKI occurred in 26.7% of patients. In these individuals, an earlier and more prominent increase in SEPP1 was observed at 4 h and 8 h, as compared with those not experiencing AKI (difference between trends *p *< 0.0001). Logistic regression analyses evidenced 4 h and 8 h SEPP1 as significantly associated with AKI (OR 1.035; 95% CI 1.002–1.068; *p* = 0.03 and 1.011; 95% CI 1.002–1.021; *p* = 0.02, respectively). ROC analyses displayed a remarkable discriminatory capacity of early SEPP1 measurements in identifying AKI (AUCs ranging from 0.682 to 0.854; *p* from 0.04 to <0.0001). In addition, 12 h-SEPP1 showed diagnostic capacity to identify patients reaching a secondary composite endpoint including major adverse kidney events (MAKEs).

**Conclusions:**

Findings from this pilot, exploratory study suggest that early SEPP1 measurement after CPB may hold great potential for improving renal risk stratification in cardiac surgery patients. Further studies in wider and more heterogeneous cohorts are needed to generalize these findings and to evaluate a possible applicability in daily practice.

## 1. Introduction

Acute Kidney Injury (AKI) remains one of the most dangerous, life-threatening complications of cardiac surgery, affecting up to 30% of patients undergoing cardiopulmonary bypass (CPB) [1, 2]. AKI amplifies the risk of postoperative mortality and morbidity, is associated with increased healthcare costs and may also drive long-term complications such as stroke, heart failure and chronic kidney disease [3]. Diagnosis of AKI usually relies on a tangible increase in serum creatinine which, however, cannot be detectable earlier than 24–48 h after the driving injury. Novel biomarkers that anticipate serum creatinine rise are therefore eagerly needed to prompt therapeutic measures for mitigating damage and preserving kidney function in a timely manner. Despite the pathogenesis of AKI is generally multifactorial, oxidative stress has recently been acknowledged as an important determining factor [4, 5]. This holds true particularly in cardiac surgery patients as prolonged cardiopulmonary bypass is a potent trigger of reactive oxygen species (ROS) and peroxidation products generation, ultimately leading to microvascular impairment and organ damage [6]. In last years, selenoprotein-p1 (SEPP1) has emerged as a key factor in the systemic responses to oxidative stress. SEPP1 is primarily secreted from liver and acts as a selenium transporter, supplying tissues and organs with this trace mineral which elicits the activity of specific glutathione peroxidase selenoenzymes (GPxs) [7]. In addition, SEPP1 seems also to be endowed with direct ROS-detoxifying capacities at the extra-cellular level [8].

Interestingly, previous studies have demonstrated an altered SEPP1 balance in rat models of AKI following cisplatin administration or ischemia/reperfusion [9], as well as in heavy-metal induced nephrotoxicity [10]. In addition, in patients undergoing CPB, increased SEPP1 levels reflect myocardial hypoxia and may predict adverse cardiovascular outcomes such as death, bradycardia or cerebral ischemia [11]. Starting from these premises, we designed an observational, pilot, hypothesis-driven study to test the possible role of SEPP1 as a predictive biomarker of AKI in the cardiac surgery setting.

## 2. Materials and Methods

### 2.1 Study Design and Patient Enrolment

198 patients consecutively referred to the Cardiac Surgery Unit of the University Hospital of Catanzaro (Catanzaro, Italy) between July 2020 and February 2021 were screened to enter in this prospective, observational study. Exclusion criteria were emergency cardiac surgery, inability to give informed consent, age <18 years, glomerular filtration rate (eGFR) <60 mL/min/1.73 $m^{2}$, acute concomitant infections, treatment with any nephrotoxic medication or contrast medium administration in the prior 2 weeks before surgery, long-term immunosuppressant therapy or a severely impaired cardiac function. The study was approved by the Local University Institutional Review Board and all subjects provided written informed consent.

### 2.2 Clinical Data and SEPP1 Measurement

Patients’ characteristics, anthropometrics, comorbidities, medications, surgical and laboratory data were recorded using a standardized case report form. Pre-operative surgical risk for mortality and renal morbidity was assessed by the short-term-score (STS) [12]. Common biochemistry tests were performed according to standard methods used in the routine clinical laboratory. Serum samples for SEPP1 measurement were obtained preoperatively and, respectively, 4 h, 8 h and 12 h after surgery. Serum samples were centrifuged at 1227 g for 15 minutes at 4 °C and the aliquots stored at –80 °C until thawed for batch analysis. All SEPP1 measurements were performed in the same laboratory (CNR-Institute of Clinical Physiology, Reggio Calabria, Italy) by a commercially available ELISA kit (Human Selenoprotein P1 ELISA kit, Cloud-Clone Corp, Houston, TX, USA).

### 2.3 Study Endpoints

The primary study endpoint was the occurrence of in-hospital post-operative AKI. This was defined according to the Kidney Disease Improving Global Outcomes (KDIGO) Clinical Practice Guidelines for Acute Kidney Injury as an increase in post-operative serum creatinine ≥0.3 mg/dL within 48 h or an increase >1.5-fold during the 7 days following surgery. The severity of AKI was also staged according to the same guidelines [13]. We also explored a secondary, pilot endpoint represented by the occurrence of major adverse kidney events (MAKEs). MAKEs were defined as a composite of death, acute worsening in renal function with need of dialysis support or a decrease in estimated glomerular filtration >25% of baseline, evaluated up to 30 days after surgery [14]. Post–hospital discharge information on renal function was obtained retrospectively by medical record review or using telephone interviews if needed.

### 2.4 Statistical Analysis

The statistical analysis was performed using the SPSS (version 24.0.0.0; SPSS Inc., Chicago, IL, USA) package and the MedCalc Statistical Software (version 14.8.1; MedCalc Software Ltd., Ostend, Belgium). Data were presented as mean ± SD, median [IQ range] or frequency percentage as appropriate. Differences between groups were determined by the unpaired *t*-test for normally distributed values, the Mann-Whitney U test for non-parametric values and the chi-square followed by a Fisher’s exact test for frequency distributions. One-way ANOVA with linear assumption was employed to analyze statistical variance of SEPP1 across the established time points (*p* for trend). Pairwise comparison by the Bonferroni’s test was used to check differences in SEPP1 time trends between subpopulations. The Pearson (R) correlation coefficient was employed to identify putative clinical predictors of SEPP1. Before testing correlations, all values showing a skewed distribution were log-transformed to better approximate normal distributions. Logistic regression analyses were performed to establish significant associations between the primary renal outcome (AKI) and any clinical variable which resulted different at baseline between the two study subpopulations (AKI and non-AKI patients). Receiver Operating Characteristics (ROC) analyses were employed to calculate the areas under the curve (AUCs) for SEPP1 considering AKI and MAKEs as status variables. The best cut-off values were computed by the Youden index. All results were considered significant if the *p* value was <0.05.

## 3. Results

### 3.1 Study Population Characteristics

The final study population consisted of 45 consecutive patients undergoing elective major cardiac surgery with CPB. The most frequent surgical intervention was isolated CABG (71.1%). Mean age was 65.6 ± 8 years. Patients were predominantly male (77.8%) and displayed a median BMI of 27.8 [IQR 25.9–30.9]. There was a high prevalence of diabetes (60%) with a median diabetes vintage of 7.5 yrs [IQR 1–12]. Virtually all individuals (95.6%) had previous history of cardiovascular or cerebrovascular disease. All patients showed normal renal function with mean serum creatinine values of 0.90 ± 0.19 mg/dL and a mean estimated GFR (CKD-EPI) of 91.8 ± 14.3 mL/min/1.73 $m^{2}$. Overall, ejection fraction was preserved (median 50%, IQR 45–55) while left atrial volume was, on average, normal to mildly abnormal (42.3 ± 8.27 mL/$m^{2}$). The majority of individuals was on RAS blockers (91.1%), beta-blockers (82.2%) and statins (84.4%). Short-term scores for risk of mortality and renal failure were low on average, with the median being 1.13 [IQR 0.70–2.40] and 1.27 [IQR 0.75–1.99], respectively. Baseline circulating SEPP1 in the whole population was 69 [IQR 39–85] ng/mL. Table 1 summarizes the main anthropometric, clinical and laboratory data of the study cohort.

**Table 1.** **Main clinical and laboratory characteristics of the study cohort and differences between subgroups of patients who developed AKI or not. Statistical differences are highlighted in bold**.

		All	AKI	no-AKI	*p*
		n = 45	n = 12	n = 33	
Patients’ characteristics					
	Age (yrs)	65.6 ± 8	64.1 ± 7.9	64.9 ± 8	0.93
	Gender (% Male)	77.8	75	78.8	0.78
	BMI (kg/$m^{2}$)	27.8 [25.9–30.9]	32.5 ± 8.2	27.8 [25.9–29.8]	0.16
	Smoking (%)	35.5	16.7	42.4	0.52
	CV disease (%)	95.6	91.7	97	0.98
	Hypertension (%)	73.3	66.7	75.7	0.39
	Diabetes (%)	60	66.7	57.6	0.73
	Diabetes vintage (yrs)	7.5 [1–12]	7 [3–11.5]	7.5 [1–12]	0.89
	NYHA class (% 1/2/3)	15.6/64.4/20	0/66.7/33.3	21.2/63.6/15.2	0.55–0.82
	Ejection fraction (%)	50 [45–55]	50.4 ± 6.1	49.6 ± 9	0.69
	**Left atrial volume (mL/$m^{2}$)**	**42.3 ± 8.27**	**46.3 ± 6.7**	**40.4 ± 8.4**	**0.04**
	Creatinine (mg/dL)	0.90 ± 0.19	0.93 ± 0.20	0.90 ± 0.20	0.81
	eGFR CKD–EPI (mL/min/$m^{2}$)	91.8 ± 14.3	91 ± 14.7	92.1 ± 14.3	0.77
	Urea (mg/dL)	39 [31.7–47.2]	42.5 [32.5–52.5]	39 [31.7–46.2]	0.21
	**Haemoglobin (g/dL)**	**12.8 ± 1.5**	**12.6 ± 1.5**	**13.8 ± 1.6**	**0.04**
	Haematocrit (%)	38.7 ± 4.7	40.4 ± 5.8	38.1 ± 4.2	0.36
	Total cholesterol (mg/dL)	142.7 ± 43.1	145.7 ± 33.5	141.6 ± 46.7	0.78
	LDL cholesterol (mg/dL)	80 [62.2–108.7]	88.9 ± 28.3	80 [60.5–112.2]	0.89
	Triglycerides (mg/dL)	102 [86–131]	97.5 [79.5–126.5]	104 [86–132]	0.75
	CK–MB (UI/L)	1.6 [1.3–2.4]	1.85 [1.50–2.20]	1.6 [1.2–2.42]	0.92
	Hs–cTN (ng/L)	16 [9.3–26.6]	15.8 [13.2–25.6]	16.6 [9.1–28.8]	0.85
	Myoglobin (nmol/L)	29 [22.5–45.7]	29.5 [26.5–37.5]	28 [21–48]	0.77
Pre-operative medications					
	ACEi/ARBs (%)	91.1	83.3	90.9	0.66
	Diuretics (%)	44.4	41.6	45.4	0.70
	Beta-blockers (%)	82.2	83.3	81.8	1.00
	Calcium channel blockers (%)	15.5	16.7	15.1	0.84
	Statins (%)	84.4	83.3	84.5	0.92
	Platelet inhibitors (%)	28.9	25	30.3	0.89
Surgical characteristics					
	Type of surgery				
	CABG only (%)	71.1	41.7	81.8	0.33
	CABG plus valve (%)	15.6	25	12.1	0.45
	Valve only (%)	11.1	25	6.1	0.29
	Other (%)	2.2	8.3	0	0.58
	Pre-operative SBP (mmHg)	130.1 ± 15.7	129 [120.5–132.5]	130.6 ± 16.2	0.76
	Pre-operative DBP (mmHg)	74.6 ± 10.9	74.6 ± 11.3	74.6 ± 10.9	0.91
	**STS renal failure score**	**1.27 [0.75–1.99]**	**3.09 ± 1.12**	**1.08 [0.58–1.62]**	**0.04**
	STS mortality score	1.13 [0.70–2.40]	2.55 ± 1.05	1.02 [0.53–1.62]	0.18
	**Cross-clamp time (min)**	**72 [56–104.2]**	**103.8 ± 33.1**	**69.1 ± 23.5**	**0.0003**
	**CPB time (min)**	**105 [91–137]**	**147.6 ± 59.3**	**104.7 ± 30.6**	**0.002**
SEPP1 measurement					
	Baseline SEPP1 (ng/mL)	69 [39–85]	69 [39–98.5]	69 [39–85]	0.98
	**SEPP1 4 h post CBP (ng/mL)**	**119 [39–414.7]**	**546.5 [260.5–1000]**	**52 [39–233.2]**	**0.0003**
	**SEPP1 8 h post CBP (ng/mL)**	**906 [279–535.5]**	**1959 [1055.5–5303]**	**628 [437.7–1254.5]**	**0.003**
	SEPP1 12 h post CBP (ng/mL)	3263 [1886.2–5042.7]	3154.5 [1799.5–5740.5]	3263 [1886.2–4696.7]	0.70

Legend: BMI, body mass index; CABG, coronary artery bypass graft; CK-MB, creatine-kinase MB; CPB, cardiopulmonary bypass; CV, cardiovascular; DBP, diastolic blood pressure; eGFR, estimated glomerular filtration rate; Hs-cTN, Highly sensitive c-troponin; LDL, low density lipoprotein; NYHA, New york health association; SBP, systolic blood pressure; SEPP1, selenoprotein-p1; STS, short-term risk.

### 3.2 SEPP1 Levels After Cardiac Surgery

Surgery was successful and well tolerated in all patients with no major complications or adverse events reported. The median cross-clamp time was 72 [IQR 56–104.2] mins while the median CPB duration was 105 [IQR 91–137] mins. SEPP1 displayed in all patients an increasing trend from baseline to 12 h after the surgical procedure (69 [IQR 39–85] to 3263 [IQR 1886.2–5042.7] ng/mL; *p* for trend <0.0001). More in detail, SEPP1 levels increased 4 h after surgery by a median of 1.66 [IQR 1–5.01] folds and further increased by 5.35 [IQR 2.55–15.2] folds from 4 h to 8 h and by 3.42 [IQR 2.27–5.34] folds from 8 h to 12 h after surgery (all *p *< 0.0001). The overall pre-post surgery (baseline to 12 h) delta increase resulted as high as 53.1 [IQR 33.4–94.6] folds (*p *< 0.0001).

**Fig. 1.**
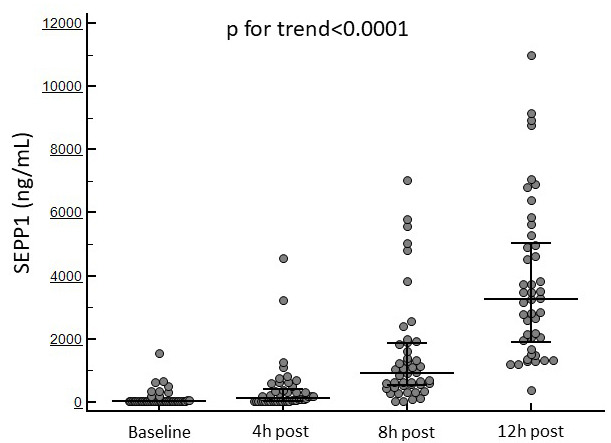
**Changes in circulating SEPP1 at the established time points in the whole study cohort**.

Fig. 1 displays the temporal trend in SEPP1 levels in the whole study population.

**Fig. 2.**
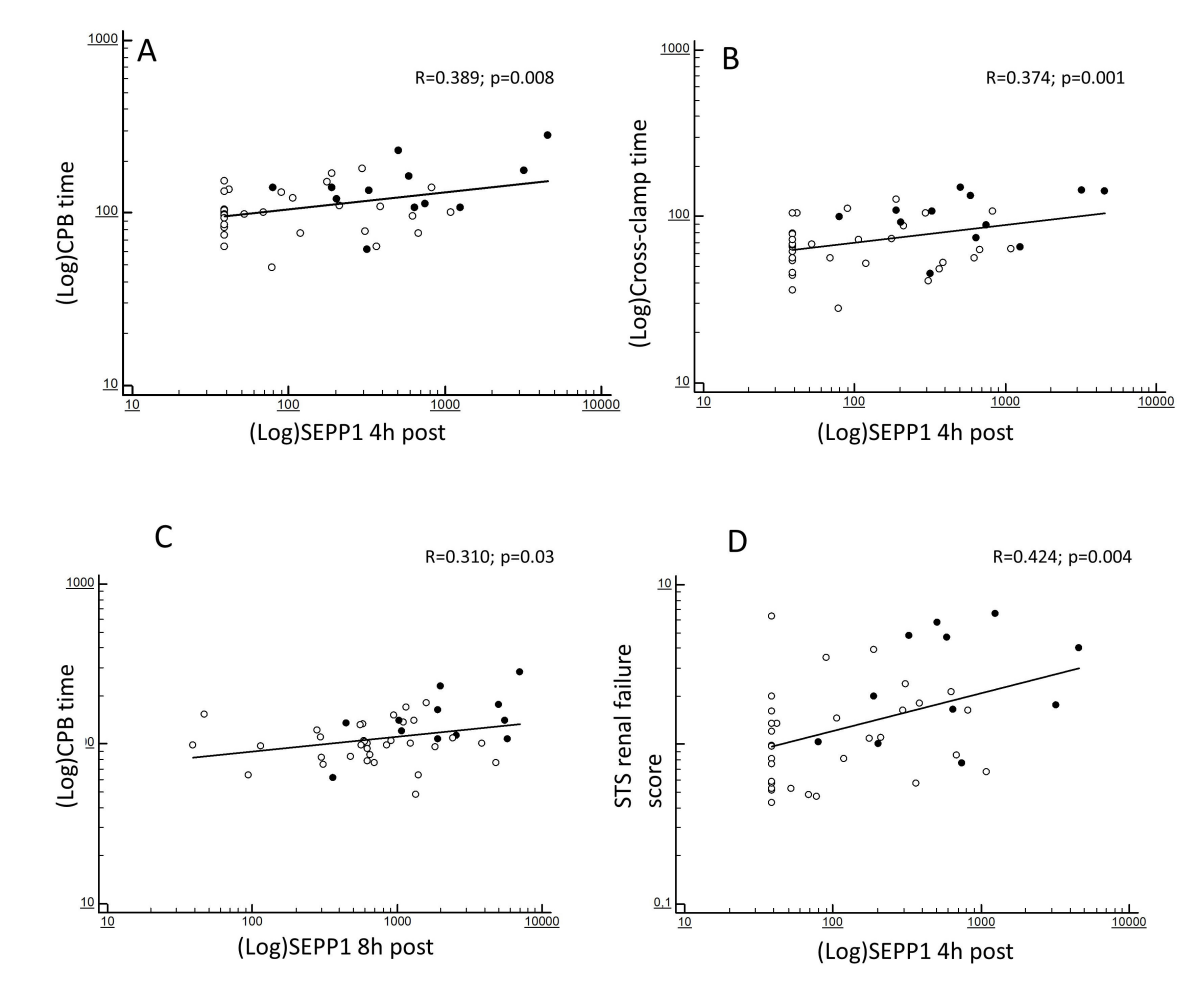
**Bivariate correlations between (log-transformed) SEPP1 measured at different time-points**. 4 h post-surgery and (log-transformed) CPB time (A), (log-transformed) cross-clamp time (B) and STS score for AKI (D) and between (log-transformed) SEPP1 measured at 8 h post-surgery and (log-transformed) CPB time (C). Black and white dots indicate patients with or without following AKI.

At correlation analyses, the increased 4 h SEPP1 levels were strongly predicted by CPB (R = 0.389; *p* = 0.008; Fig. 2A) and cross-clamp duration (R = 0.374; *p* = 0.001; Fig. 2B). In addition, such levels reflected the severity of the baseline ST renal failure score (R = 0.424; *p* = 0.004; Fig. 2D). 8 h SEPP1 levels were also significantly predicted by CPB time (R = 0.310; *p* = 0.03; Fig. 2C) but no associations were apparently found with cross-clamp duration (R = 0.186; *p* = 0.35).

### 3.3 Renal Outcomes After Cardiac Surgery

The overall incidence of post-surgery AKI was 26.7% (n = 12). Stage 1 AKI occurred in four patients (33.3%), stage 2 AKI occurred in seven (58.3%) and stage 3 AKI in one patient (8.3%). At baseline, patients who developed AKI displayed a significantly increased left atrial volume, lower haemoglobin levels and a worsen STS renal failure (all *p* = 0.04) as compared with those without AKI. Overall cross-clamp and CPB times were also significantly longer in AKI patients (*p* = 0.0003 and 0.002, respectively). No differences in other clinical, surgical, anthropometric or laboratory parameters were noticed (Table 1). The study secondary exploratory MAKE endpoint occurred in seven (15.7%) patients. Of these, one patient died while the remaining six experienced a reduction in eGFR which did not revert to within 25% of baseline values. None of them necessitated dialysis support. The median days to available postoperative data on renal function was 18 [IQR 11–26]. The MAKE outcome was apparently more frequent in patients who previously suffered from post-surgery AKI (33.3% vs. 21.2%).

### 3.4 SEPP1 Levels in Patients with or without Following AKI

**Fig. 3.**
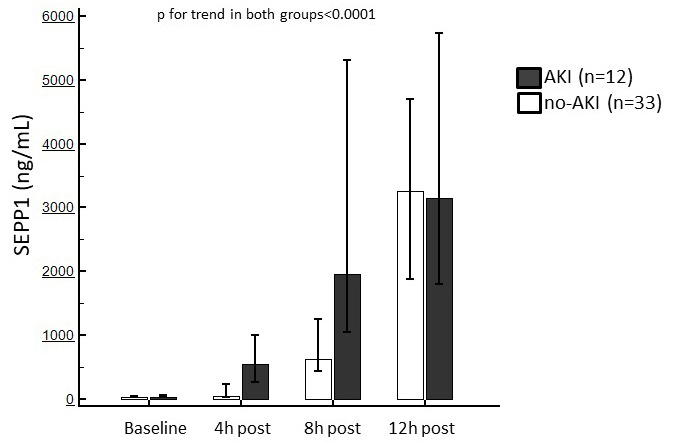
**Median of circulating SEPP1 measured at the established time points in patients with or without following AKI**. Trends in both sub-population were statistically significant.

Baseline SEPP1 levels were similar between patients with or without following AKI (69 [IQR 39–98.5] vs. 69 [IQR 39–85] ng/mL). After surgery, a significant increase in circulating SEPP1 was observed in both sub-populations (*p *< 0.0001) but the two trends were statistically different (*p* = 0.001). Indeed, the rise in circulating SEPP1 manifested earlier in AKI patients (4 h SEPP1 546.5 [IQR 260.5–1000] vs. 52 [IQR 39–233.2] ng/mL, *p* = 0.0003; 8 h SEPP1 1959 [IQR 1055.5–5303] vs. 628 [IQR 437.7–1254.5] ng/mL, *p* = 0.003 in AKI vs. non-AKI, respectively). Conversely, no difference in SEPP1 measured 12 h after surgery was noticed between the two subgroups (*p* = 0.70) (Fig. 3).

### 3.5 Clinical Predictors of AKI 

All variables which were different at baseline between AKI and non-AKI patients were tested against the primary renal endpoint by logistic regression analysis. In order to avoid co-linearity, we built two different models including 4 h and 8 h SEPP1 separately. The first model confirmed increased 4 h SEPP1 and cross-clamp duration as significant predictors of AKI (OR 1.035, 95% CI 1.002–1.068; *p* = 0.03 and OR 3.119, 95% CI 1.001–10.466; *p* = 0.04, respectively). Conversely, no significant associations with the renal endpoint were described for left atrial volume, CPB time and haemoglobin levels. Likewise, SEPP1 measured 8 h after CPB (OR 1.011, 95% CI 1.002–1.021; *p* = 0.02) and cross-clamp time (OR 4.653, 95% CI 1.109–19.526; *p* = 0.03) remained the sole variables significantly associated with AKI also in the second model. Table 2 summarizes findings at logistic regression analyses.

**Table 2.** **Logistic regression analysis of clinical predictors of AKI. (Model A) including SEPP1 measured 4 h post CPB; (Model B) including SEPP1 measured 8 h post CPB. Statistically significant associations are highlighted in bold**.

Model A	Unit of increase	OR	95% CI	*p*
**Cross-clamp time**	**10 mins**	**3.119**	**1.001–10.466**	**0.04**
**SEPP1 4 h post CPB**	**10 ng/mL**	**1.035**	**1.002–1.068**	**0.03**
Left atrial volume	1 mL/$m^{2}$	1.402	0.920–2.134	0.12
CPB time	10 mins	0.711	0.307–1.644	0.42
Haemoglobin	1 g/dL	2.181	0.874–5.439	0.09
Model B		OR	95% CI	*p*
**Cross-clamp time**	**10 mins**	**4.653**	**1.109–19.526**	**0.03**
**SEPP1 8 h post CPB**	**10 ng/mL**	**1.011**	**1.002–1.021**	**0.02**
Left atrial volume	1 mL/$m^{2}$	1.107	0.968–1.265	0.14
CPB time	10 mins	0.565	0.244–1.305	0.18
Haemoglobin	1 g/dL	2.036	0.717–5.780	0.11

Legend: CPB, cardiopulmonary bypass; SEPP1, selenoprotein-p1; STS, short-term risk.

### 3.6 Diagnostic Performance of SEPP-1 in Identifying Renal Outcomes

Two different models of ROC analysis were built to test the diagnostic capacity of SEPP1 with respect to the primary renal outcome (AKI). In the first model (Table 3), we tested absolute SEPP1 values measured at the various time points. Baseline and 12 h SEPP1 had no or limited diagnostic power in this respect, showing an Area Under the Curve (AUC) of 0.501 (95% CI 0.354–0.649) and 0.538 (95% CI 0.330–0.746), respectively. Conversely, SEPP1 measured 4 h after CPB displayed a remarkable diagnostic capacity with an AUC of 0.854 (95% CI 0.743–0.964). The best discriminatory cut-off value was 178 ng/mL, yielding a sensitivity of 91.6% (95% CI 61.5–99.8) and a specificity of 69.7% (95% CI 51.3–84.4). A good diagnostic performance was also described for 8h SEPP1, showing an AUC of 0.790 (95% CI 0.620–0.961) and a best threshold of 1840 ng/mL with a sensitivity of 66.6% (95% CI 34.9–90.1) and a specificity of 90.9% (75.7–98.1).

**Table 3.** **Areas under the curve (AUCs) and best cut-off values (Youden index) of absolute circulating SEPP1 measured at different time points to detect patients with following AKI. Statistically significant AUCs are highlighted in bold**.

	AUC [95% CI]	*p*	Best cut-off (ng/mL)	Sens.%	Spec.%
Baseline SEPP1	0.501 [0.354–0.649]	0.98	≤195	91.6 [61.5–99.8]	18.1 [7.0–35.54]
**SEPP1 4 h post CPB**	**0.854 [0.743–0.964]**	< **0.0001**	> **178**	**91.6 [61.5–99.8]**	**69.7 [51.3–84.4]**
**SEPP1 8 h post CPB**	**0.790 [0.620–0.961]**	**0.0009**	> **1840**	**66.6 [34.9–90.1]**	**90.9 [75.7–98.1]**
SEPP1 12 h post CPB	0.538 [0.330–0.746]	0.72	>4959	41.6 [15.2–72.3]	81.8 [64.5–93.0]

Legend: CPB, cardiopulmonary bypass; SEPP1, selenoprotein-p1.

In the second model (Table 4), we considered a two-point delta change in SEPP1 levels between all possible time point combinations. With this approach, a 4.5 folds increase in circulating SEPP1 from baseline to 4 h after CPB yield the best diagnostic capacity in diagnosing patients with following AKI with an AUC of 0.843 (95% CI 0.683–1.000; *p *< 0.0001), a sensitivity of 75.0% (95% CI 42.8–94.5) and a specificity of 87.8% (95% CI 71.8–96.6). A statistically significant, although less remarkable discriminatory capacity was observed for all the other time points combinations (AUCs spanning from 0.813 to 0.683; *p* ranging from <0.0001 to 0.04), with delta changes from baseline to 12 h post-CPB being the only exception (AUC 0.528; *p* = 0.78).

**Table 4.** **Areas under the curve (AUCs) and best cut-off values (Youden index) of delta changes in circulating SEPP1 to detect patients with following AKI. Statistically significant AUCs are highlighted in bold**.

	AUC [95% CI]	*p*	Best cut-off (fold increase)	Sens.%	Spec.%
△ ** baseline-4 h post CPB**	**0.843 [0.683–1.000]**	< **0.0001**	> **4.5**	**75.0 [42.8–94.5]**	**87.8 [71.8–96.6]**
△ ** baseline-8 h post CPB**	**0.755 [0.574–0.936]**	**0.005**	> **24.5**	**66.6 [34.9–90.1]**	**84.8 [68.1–94.9]**
△ ** 4 h post-8 h post CPB**	**0.682 [0.503–0.861]**	**0.04**	≥ **5.4**	**91.7 [61.5–99.8]**	**57.6 [39.2–74.5]**
Δ baseline-12 h post CPB	0.528 [0.336–0.720]	0.78	>33.3	83.3 [51.6–97.9]	30.3 [15.6–48.7]
△ ** 4 h post-12 h post CPB**	**0.813 [0.677–0.949]**	< **0.0001**	≥ **17.6**	**91.7 [61.5–99.8]**	**69.7 [51.3–84.4]**
△ ** 8 h post-12 h post CPB**	**0.793 [0.619–0.967]**	**0.001**	≥ **3.2**	**83.3 [51.6–97.9]**	**66.7 [48.2–82.0]**

Legend: CPB, cardiopulmonary bypass; SEPP1, selenoprotein-p1.

**Fig. 4.**
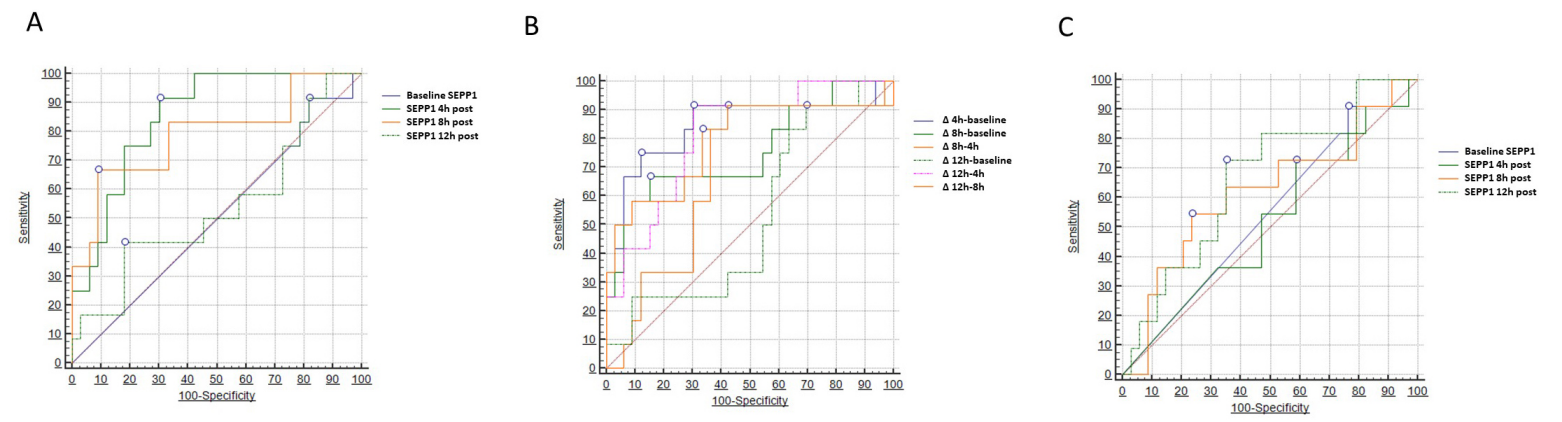
**Areas under the curve (AUCs) for circulating SEPP1–**AUCs for (A) SEPP1 measured at different time-points and (B) Δ changes in SEPP1 (n/folds increase) to detect patients with following AKI; (C) SEPP1 at different time-points to identify patients with following major kidney adverse events (MAKEs).

On the contrary, SEPP1 displayed only marginal diagnostic capacities with respect to the correct identification of patients experiencing the secondary MAKE renal endpoint. To this end, only SEPP1 measured 12 h after CPB demonstrated a limited, although statistically significant discriminatory ability with an AUC of 0.663 (95% CI 0.508–0.848, *p* = 0.05) and an optimal threshold of 2829 ng/mL, yielding a sensitivity of 72.7% (95% CI 39.1–94.0) and a specificity of 64.7% (95% CI 46.5–80.3). Conversely, all the other time-measurements appeared to be not discriminatory in this regard. Fig. 4 depicts all findings from the three different ROC analyses (Table 5).

**Table 5.** **Areas under the curve (AUCs) and best cut-off values (Youden index) of circulating SEPP1 measured at different time points to detect patients developing MAKEs. Statistically significant AUCs are highlighted in bold**.

	AUC [95% CI]	*p*	Best cut-off (ng/mL)	Sens.%	Spec.%
Baseline SEPP1	0.541 [0.397–0.685]	0.57	>78	90.9 [58.7–99.8]	23.5 [10.7–41.2]
SEPP1 4 h post CPB	0.516 [0.316–0.716]	0.87	≥212	72.7 [39.0–94.0]	41.2 [24.6–59.3]
SEPP1 8 h post CPB	0.618 [0.408–0.827]	0.27	>578	54.5 [23.4–83.3]	76.5 [58.8–89.3]
**SEPP1 12 h post CPB**	**0.663 [0.508–0.848]**	**0.05**	> **2829**	**72.7 [39.1–94.0]**	**64.7 [46.5–80.3]**

Legend: CPB, cardiopulmonary bypass; SEPP1, selenoprotein-p1.

## 4. Discussion

Major cardiac surgery portends an exceedingly high risk for severe clinical complications related to AKI. This assumption mostly relies on two key reasons. First the CPB procedure itself is known to elicit or worsen renal damage through various mechanisms such as renal ischemia and reperfusion, thromboembolism, hemolysis, inflammation and oxidative stress [2]. Second, the clinical diagnosis of AKI is usually delayed 2 to 3 days after the true AKI onset as glomerular filtration rate must decline significantly before serum creatinine accumulates in the blood. No less important, postoperative haemodilution could mask creatinine elevation, while urine output monitoring for AKI detection may lack of sensitivity due to the frequent post-operatory use of diuretics. Such a delayed identification may hamper decision making and optimization of post-operative care, therefore increasing mortality, morbidity and the length of hospital stay.

Results from our pilot study point at SEPP1 as a novel candidate biomarker for early AKI risk stratification in this population setting. First of all, as reported by previous observations [11], we found a remarkable increase in SEPP1 levels after CPB in the whole study cohort, with circulating values peaking up to 53 fold 12 h after surgery. We may speculate that such a significant increase in SEPP1 may represent part of a compensatory response to a systemic oxidative stress induced by the extracorporeal procedure [6, 15]. The close relationship between CPB and SEPP1 increase was further supported by correlation analyses, which demonstrated a significant impact of ischemia duration particularly on the first (early), 4 to 8 h rise in the circulating levels of this biomarker.

The key findings of our study, however, pertain to the different trend in SEPP1 levels observed in patients in whom post-surgery AKI occurred. In this respect, AKI patients displayed an earlier (4–8 h) and more prominent increase in SEPP1 as compared to others, while such levels reached comparable values at later measurements. The close connection between AKI occurrence and early SEPP1 response was further confirmed by logistic regression analyses, in which early SEPP1 measurements remained significantly associated with a growing risk of AKI unlike other relevant clinical parameters. We cannot clarify the biological meaning of the particular pattern of SEPP1 response in relationship with post-surgery AKI. Under normal conditions, circulating SEPP1 distributes selenium to tissues in proportion to the cellular expression of the apolipoprotein-E receptor-2 (Apo-ER2), which also serves as SEPP1 receptor. Such release can be increased in conditions of oxidative stress, which triggers an increased Apo-ER2 expression [16]. At the proximal renal tubule level, however, SEPP1 also binds megalin, another surface receptor which expression can be upregulated by local damage [17]. Mechanistic studies are needed to ascertain whether the more prominent rise in SEPP1 levels observed in AKI patients reflects an increased local demand or the acquired capacity of the kidney to synthesize this protein to better sustain tubular damage. Nevertheless, in our study cohort SEPP1 demonstrated a clear diagnostic capacity while tested on the established renal endpoint. Predictably, discrimination was effective for early measurements (4 to 8 h) and was also remarkable when considering a two-point delta change instead of absolute values, similarly to what observed for other AKI biomarkers [18]. Interestingly, as alluded to before, late measurements (12 h) were not discriminant with respect to AKI occurrence. Conversely, these held a limited although significant capacity in identifying individuals experiencing major adverse kidney events after hospital discharge. Such a further application of SEPP1 dosage would deserve appropriate future investigations as it may help improving risk stratification of chronic kidney disease following in-hospital AKI, a condition that remains, unfortunately, rather frequent and difficulty predictable [19].

Our study has some strengths and weaknesses that deserve mentioning. Strengths include a prospective design with repeated time measurements, an universally validated renal endpoint which occurred in a substantial percentage of subjects and a thorough analytical approach focusing on single as well as multiple time-point analyses, to better define the discriminatory profile of SEPP1. The main weakness is the small sample size which limited the possibility of more in-depth analyses and may have underpowered the study against the secondary composite endpoint. No less important, the cohort was relatively homogeneous in terms of comorbidities, types of surgery and did not include patients with a pre-existing impaired renal function. Given the observational nature of the study, the presence of selection bias and significant residual confounding cannot thus be fully ruled out. Finally, SEPP1 was measured up to 12 h after surgery; although previous evidence suggests that SEPP1 levels revert to normal by 24 h after CPB [11], an extended observation would have been more helpful in characterizing the dynamic relationship between SEPP1 and serum creatinine increase.

## 5. Conclusions

Early SEPP1 measurement after CPB may hold great potential for identifying cardiac surgery patients at risk of developing in-hospital AKI. However, the findings made in the present study must be considered only preliminary and need to be generalized in wider and more heterogeneous cohorts. Focused investigations are also advocated to clarify the exact biological role of SEPP1 in counteracting renal, as well as systemic, oxidative stress.
